# Improvement of Strength-Toughness-Hardness Balance in Large Cross-Section 718H Pre-Hardened Mold Steel

**DOI:** 10.3390/ma11040583

**Published:** 2018-04-10

**Authors:** Hanghang Liu, Paixian Fu, Hongwei Liu, Dianzhong Li

**Affiliations:** 1 Institute of Metal Research, Chinese Academy of Sciences, 72 Wenhua Road, Shenyang 110016, China; hhliu15b@imr.ac.cn (H.L.); hwliu@imr.ac.cn (H.L.); 2 School of Materials Science and Engineering, University of Science and Technology of China, 72 Wenhua Road, Shenyang 110016, China

**Keywords:** 718H pre-hardened mold steel, heat treatments, strength-toughness-hardness, microstructure

## Abstract

The strength-toughness combination and hardness uniformity in large cross-section 718H pre-hardened mold steel from a 20 ton ingot were investigated with three different heat treatments for industrial applications. The different microstructures, including tempered martensite, lower bainite, and retained austenite, were obtained at equivalent hardness. The microstructures were characterized by using metallographic observations, scanning electron microscopy (SEM), transmission electron microscopy (TEM), X-ray diffraction (XRD), and electron back-scattered diffraction (EBSD). The mechanical properties were compared by tensile, Charpy U-notch impact and hardness uniformity tests at room temperature. The results showed that the test steels after normalizing-quenching-tempering (N-QT) possessed the best strength-toughness combination and hardness uniformity compared with the conventional quenched-tempered (QT) steel. In addition, the test steel after austempering-tempering (A-T) demonstrated the worse hardness uniformity and lower yield strength while possessing relatively higher elongation (17%) compared with the samples after N-QT (14.5%) treatments. The better ductility of A-T steel mainly depended on the amount and morphology of retained austenite and thermal/deformation-induced twined martensite. This work elucidates the mechanisms of microstructure evolution during heat treatments and will highly improve the strength-toughness-hardness trade-off in large cross-section steels.

## 1. Introduction

The development of the mold industry has promoted the production and consumption as well as the research of large cross-section 718H pre-hardened mold steel [1,2]. Pre-hardened mold steel, such as the medium-carbon low-alloyed steel family AISI P20 and its derived varieties DIN 1.2738 (German grade) and 718 (Swedish grade), has been widely used in industry. Usually, the AISI P20 family is known as quenching and tempering (QT) pre-hardened steel for plastic mold, because it is delivered as a QT condition from the steelworks to the mold producers and needs no further heat treatment after a mold has been machined [3]. Manufacturers only need to rough and finish it by a milling and grinding processes. For present complex geometries, novel Super Abrasive Machining (SAM) is presented as a solution and can be extended to hardened steels [4]. Therefore, our research needs to focus on the heat treatment process before the mold steel is shipped. In the actual production process, the range of hardness of the pre-hardened mold steel should be controlled between 32 and 40 HRC. Simultaneously, toughness, ductility, wear resistance, and polishability properties are essential in pre-hardened mold steel to prevent deformation during service [5,6]. In recent years, large-section pre-hardened mold steel with a thickness exceeding 800 mm was developed in response to fierce competition surrounding technology and cost. Simultaneously, the conventional QT treatment for pre-hardening after forging was used to prevent hardness fluctuation [7]. However, in most cases, the tempered martensite microstructure produced by QT treatment contains coarse chain carbides, and results in poor hardness uniformity across large cross-sections of pre-hardened mold steel [6].

To date, there has been little research on the 718H pre-hardened mold steel. The influence of microstructure on fracture toughness properties [8], and tensile as well as fatigue properties of large 718H heat-treated mold steels were studied by Firrao [9]. In addition, Hoseiny investigated the microstructures in continuous-cooled and quench-tempered pre-hardened mold steel [10], as well as the effect of heat treatment on the microstructure and machinability of pre-hardened mold steel [6]. At the same time, the optimal tempering temperature of 718H pre-hardened mold steel had been determined in the range of 530 to 560 °C by our previous research [11].

It is also well known that spheroidizing of carbides would improve the machinability of mold steel [6]. Therefore, a suitable heat treatment that brings finely spheroidized carbides after tempering may be beneficial to mechanical properties compared with the conventional QT heat treatment. In addition, due to the characteristic of 718H pre-hardened mold steel with large cross-section, conventional techniques such as thermo-mechanical treatment or accelerated cooling are not available for grain refinement to improve toughness. However, it is likely to increase toughness levels of large forging steel through grain refinement by normalizing, resulting in the excellent combination of impact and tensile properties [12]. Simultaneously, Tu [13] reported that lower bainite in a JIS SK5 tool steel showed a higher toughness and tensile strength than tempered martensite at equivalent hardness.

In this study, the 718H pre-hardened mold steel from a 20 ton ingot has been subjected to normalizing prior to QT treatment. In addition, austempering and tempering (A-T) is taken as a new heat treatment process to obtain lower bainite microstructure at equivalent hardness compared with the conventional QT heat treatment. The strength-toughness combination and hardness uniformity after different heat treatments are compared in order to improve the comprehensive mechanical properties of large cross-section 718H pre-hardened mold steel. In addition, the mechanisms referring to mechanical properties are confirmed by experiments and theoretical analysis.

## 2. Experimental Procedures

### 2.1. Materials and Heat Treatment

**Figure 1:**
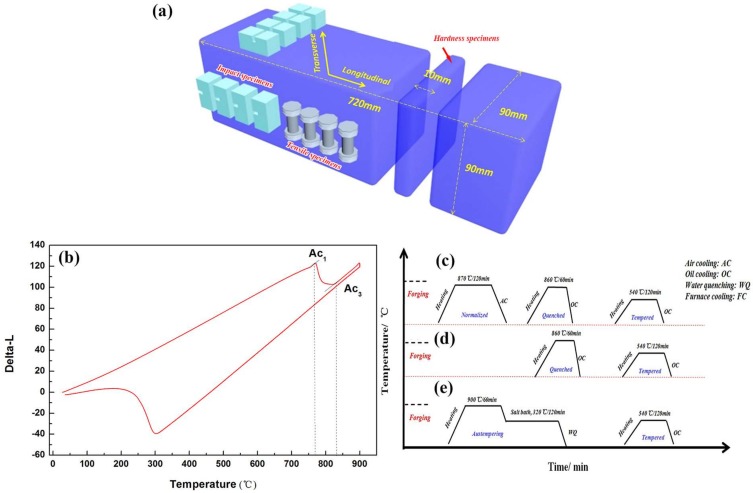
(**a**) Sampling position for mechanical properties tests, (**b**) The dilatometric curve of test steel; Schematic illustration of the different heat treatments (**c**) N-QT, (**d**) QT, and (**e**) A-T.

A 20 ton ingot of 718H pre-hardened mold steel was smelt in electric arc furnace, followed by ladle furnace (LF) refining and vacuum degassing (VD). The chemical composition is shown in Table 1. A round piece with a thickness of 180 mm was cut by the band saw at half the length of the ingot, and then a rectangular sample with the dimensions of 180 mm × 180 mm × 180 mm was obtained at 1/2 of the radius. The rectangular as-cast sample was forged at the temperature ranging from 950 to 1150 °C, and the final size was 90 mm × 90 mm × 720 mm, as shown in Figure 1a (unit: mm). In addition, all specimens for mechanical properties tests were roughly machined according to the standard procedure prior to heat treatment. Stress-relieving annealing (heated through to 550 °C, holding time 2 h. Cool slowly to room temperature) is essential for large cross-section 718H pre-Hardened Mold Steel due to the great machining stress after roughing. However, in our study, Stress-relieving annealing was not adopted because of the smaller size of the test sample. In addition, the temperature-dependent dilatometric curve of the test steel is shown in Figure 1b, which indicates that the Ac_1_ (austenization starting point) and Ac_3_ (austenization ending point) were 764 °C, 829 °C, respectively, measured by the tangent method. In addition, the heat treatment processes are as follows:

Normalizing, quenching, and tempering (N-QT treatment), consisting of normalizing at 870 °C for two hours, followed by quenching in an oil bath at 860 °C and subsequent tempering at 540 °C for two hours according to our previous research [11], as shown in Figure 1c.

Quenching and tempering (QT treatment), consisting of holding at 860 °C for one hour followed by oil cooling, and subsequent tempering at 540 °C for two hours, as shown in Figure 1d.

Austempering and tempering (A-T treatment), consisting of austenization at 900 °C for one hour, isothermal holding in salt bath at 320 °C for two hours, and subsequent tempering at 540 °C for two hours, as shown in Figure 1e.

### 2.2. Microstructure Observation

The microstructure characteristics of the specimens after different heat treatments were examined with metallographic observations (Leica Co, DM ILM, Wetzlar, Germany), scanning electron microscopy (SEM) (JSM-6301F, Japan Electronics Corporation, Tokyo, Japan), and transmission electron microscopy (TEM). In addition, the grain size of the specimens was statistically analyzed by using Image-Pro Plus 6.0 software (Media Cybernetics Inc., Rockville, MD, USA) and electron back-scattered diffraction (EBSD) under OM conditions. EBSD were performed on carefully polished specimens to diminish any surface stress. In addition, the volume fractions of retained austenite (*V_RA_*) and the dislocation density were determined by X-ray diffraction (XRD) with Cu-K*α* (*λ* = 1.5406 Å) radiation using an X-ray diffract-meter. The direct comparison method of the integrated intensity of the austenite peaks, (2 0 0)*_γ_*, (2 2 0)*_γ_*, and (3 1 1)*_γ_*, and the martensite peaks, (2 0 0)*_α_* and (2 1 1)*_α_*, were used for calculating retained austenite based on the method described in [14,15,16] with Equation (1):

(1) $$V\gamma =\frac{\frac{I_{\gamma }^{\left(220\right)}}{I_{\gamma }^{\left(220\right)}+G_{1}\cdot I_{\alpha }^{\left(200\right)}}+\frac{I_{\gamma }^{\left(220\right)}}{I_{\gamma }^{\left(220\right)}+G_{2}\cdot I_{\alpha }^{\left(211\right)}}+\frac{I_{\gamma }^{\left(200\right)}}{I_{\gamma }^{\left(200\right)}+G_{3}\cdot I_{\alpha }^{\left(200\right)}}+\frac{I_{\gamma }^{\left(200\right)}}{I_{\gamma }^{\left(200\right)}+G_{4}\cdot I_{\alpha }^{\left(211\right)}}+\frac{I_{\gamma }^{\left(311\right)}}{I_{\gamma }^{\left(311\right)}+G_{5}\cdot I_{\alpha }^{\left(200\right)}}+\frac{I_{\gamma }^{\left(311\right)}}{I_{\gamma }^{\left(311\right)}+G_{6}\cdot I_{\alpha }^{\left(211\right)}}}{6}$$

The phase intensities, $I_{\alpha }^{\left(200\right)},\;I_{\alpha }^{\left(211\right)},\;I_{\gamma }^{\left(220\right)},\;I_{\gamma }^{\left(200\right)}\;\mathrm{and}\;I_{\gamma }^{\left(311\right)}$ are obtained using the integrated intensities of the (200)*_α_*, (211)*_α_*, (200)*_γ_*, (220)*_γ_* and (311)*_γ_* peaks, respectively. In addition, *G* is the factor for the austenite (*R_γ_*) and martensite (*R_α_*) peak intensities, $G_{1}=\frac{R_{\gamma }^{\left(220\right)}}{R_{\alpha }^{\left(200\right)}}=1.38,\;G_{2}=\frac{R_{\gamma }^{\left(220\right)}}{R_{\alpha }^{\left(211\right)}}=0.06,\;G_{3}=\frac{R_{\gamma }^{\left(200\right)}}{R_{\alpha }^{\left(200\right)}}=2.50,\;G_{4}=\frac{R_{\gamma }^{\left(200\right)}}{R_{\alpha }^{\left(211\right)}}=1.19,\;G_{5}=\frac{R_{\gamma }^{\left(311\right)}}{R_{\alpha }^{\left(200\right)}}=2.02,\;G_{5}=\frac{R_{\gamma }^{\left(311\right)}}{R_{\alpha }^{\left(211\right)}}=0.96\;$ [17]. The samples for XRD measurements were prepared by the electro-polishing method to prevent a phase transformation during mechanical polishing. Then, the XRD test was performed with an acceleration voltage of 50 kV, a beam current of 180 mA, under a scan step of 0.02° from 40 to 105°, using a scanning mode of 2theta/theta. The peaks and the peak intensities were indexed and calculated by MDI Jade 6.5 software (version 6.5, Materials Data Inc., Livermore, CA, USA). In addition, the substructure of the tempered carbides after different heat treatments were observed using an XRD with a scanning angle from 15 to 85° and a scanning speed of 2°/min as well as a field-emission transmission electron microscope (TEM) operated at 200 kV. The TEM observation was conducted by using F20 (FEI company, Hillsboro, OR, USA), which is equipped with an Oxford INCA type spectrometer (Japan Electronics Corporation, Tokyo, Japan) and GATAN 832 CCD image recorder (Japan Electronics Corporation, Tokyo, Japan). TEM samples were machined to the thickness of approximately 50 to 60 μm by SiC paper, then punched into disks of 3 mm in diameter and further thinned by twin-jet electro polishing, mixing in of 10 vol % perchloric acid ethanol solutions at a voltage of 25 V and a temperature between −30 and −20 °C. In addition, the morphologies of impact and tensile fractures were examined with SEM equipped with energy dispersive X-ray spectroscopy (EDX).

### 2.3. Mechanical Tests

Determinations of Vickers hardness were made using a 500 g load for 15 s, and the hardness tests were performed in a Wilson Rockwell device (LCR-500, LECO Company, St. Joseph, MI, USA) using the Rockwell C scale. The hardness value was measured 10 times on each sample to evaluate the hardness uniformity of the cross-section after different heat treatments. Charpy U-notch specimens (10 mm × 10 mm × 55 mm) were tested on a pendulum-type impact testing machine (RKP450, Zwick-Roell Company, Ulm, Germany) with a 300 J hammer. In addition, the rounds bar tensile specimens were prepared in the transverse direction with the gage length and diameter of 25 and 5 mm, respectively. Then, they were tested at a strain rate of 0.5 mm/min by using an AG-100KNG tensile machine (Shimadzu, Kyoto, Japan). At least four impact and tensile tests for each heat treatment process are adopted here for the average value. In addition, all the mechanical properties were tested at room temperature.

## 3. Results

### 3.1. Microstructure

**Figure 2:**
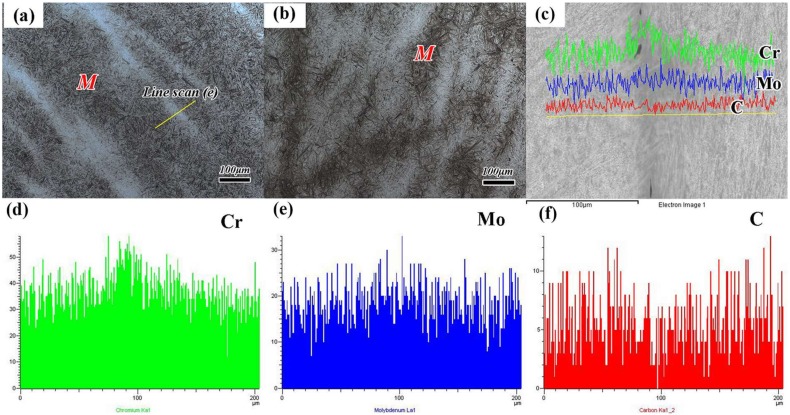
(**a**) Metallographic micrograph after normalizing, (**b**) metallographic micrograph after conventional forging (QT), (**c**) The line scan of microscopic segregation area after normalizing, (**d**) Cr segregation, (**e**) Mo segregation, and (**f**) C segregation. (The insert in Figure 2a is the position for line scan, M represents the martensite).

**Figure 3:**
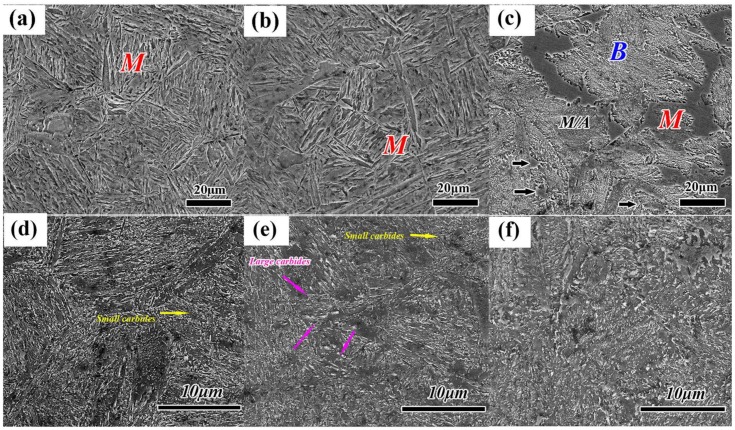
SEM observations of the samples with different heat treatments (**a**) quenched samples of N-QT, (**b**) quenched samples of QT, (**c**) quenched samples of A-T, (**d**) tempered samples of N-QT, (**e**) tempered samples of QT, and (**f**) tempered samples of A-T (M represents the martensite, B represents the bainite).

**Figure 4:**
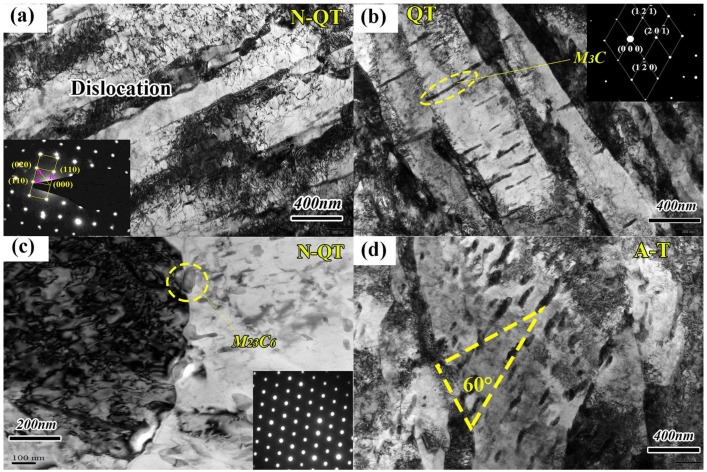
TEM observations of the tempered samples after different heat treatments (**a**) N-QT, (**b**) QT, (**c**) N-QT, and (**d**) A-T. (The insert in Figure 4a is the SAED pattern of martensite matrix, the insert in Figure 4b is the SAED pattern of M_3_C, and the insert in Figure 4c is the SAED pattern of M_23_C_6_).

Figure 2a,b shows the microstructures consisting of the martensitic matrix and some white microscopic segregation areas after normalizing and conventional forging process (QT). According to the line scan analysis in Figure 2c–f, the segregation of Cr, Mo, and C elements on the white microscopic segregation area are obvious. The sample after normalizing treatment contains fewer microscopic segregation areas compared with that of conventional forging process. The microstructures of the quenched and tempered samples after different heat treatments are systematically investigated by SEM and TEM, as shown in Figure 3 and Figure 4. The result shows that the quenched samples treated by N-QT and QT have typical lath martensite microstructures (Figure 3a,b). However, the quenched microstructure treated by A-T mainly consisting of a bainitic ferrite and martensitic bands as well as carbides. In addition, a small amount of the blocky morphology of M/A constituents (a mixture of martensite embedded in austenite [6]) is also clearly seen in Figure 3c. This is consistent with the description of the literature [2].

In addition, the tempered microstructures of samples treated by N-QT and QT have a typical tempered martensite consisting of precipitation of carbides at the lath boundaries (Figure 4a) and the strip-like carbides in the interior of the lath martensite (Figure 4b). The difference between the tempered microstructures after N-QT and QT heat treatments is mainly the morphology and distribution of the carbides. The microstructure after N-QT treatment consists of the finely spheroidized carbides which uniformly distribute in the tempered martensite matrix compared with that of QT treatment (Figure 3d). The morphology and distribution of carbides are rather homogenous after N-QT treatments compared with that of the QT treatment. However, the tempered microstructure after A-T treatment are significantly different with N-QT and QT heat treatments, which consists of the lower bainite and tempered martensite, as shown in Figure 3f. In addition, the TEM micrograph in Figure 4d shows the tempered microstructure consisting of bainitic ferrite plates and carbides within the ferrite laths at 55~60° angle against the long axis of ferrite plates, which is the typical characteristic of lower bainite [18].

**Figure 5:**
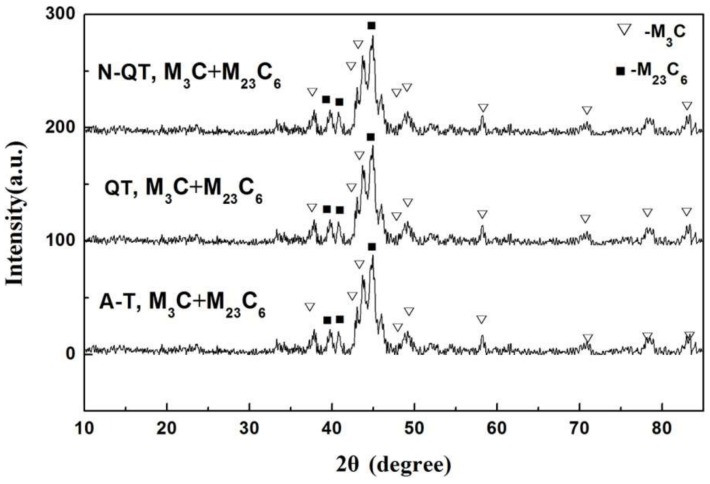
X-ray diffraction patterns of the samples after different heat treatments.

Meanwhile, the crystal structures of the tempered carbides after different heat treatments are systematically investigated by TEM bright-field (BF) observation and SAED pattern, and the results are shown in Figure 4b,c. A strip-like particle in Figure 4b is identified to be the M_3_C carbide. The particle in Figure 4c is identified to be M_23_C_6_ carbides with the face centered cubic crystal structure. The larger irregular block M_23_C_6_ carbides will be precipitated along the grain and sub-grain boundaries when the tempering temperature is above 500 °C [19]. In addition, from the XRD spectra of the tempered samples after different heat treatments in Figure 5, the tempered carbides of M_3_C and M_23_C_6_ are detected in all test steels.

**Figure 6:**
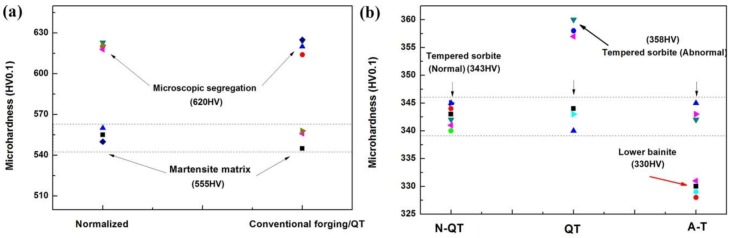
(**a**) The microhardness of different micro-constituents after different heat treatments, Normalizing, and conventional forging/QT, (**b**) the microhardness observation of the tempered microstructure after different heat treatments (N-QT, QT, and A-T).

The microhardness of different micro-constituents after different heat treatments is presented in Figure 6. The results show that the microhardness of the microscopic segregation areas in Figure 2a,b is higher than that of the matrix, and the difference can reach about 65 HV (Figure 6a). In addition, the microhardness of the tempered microstructure shows that the sample after QT treatment has a certain fluctuation of microhardness. The total deviation can reach about 15 HV. Meanwhile, the microhardness of lower bainite after A-T treatment is about 13 HV lower than that of normal tempering martensite (340~345 HV).

### 3.2. Grain Structure and Retained Austenite

**Figure 7:**
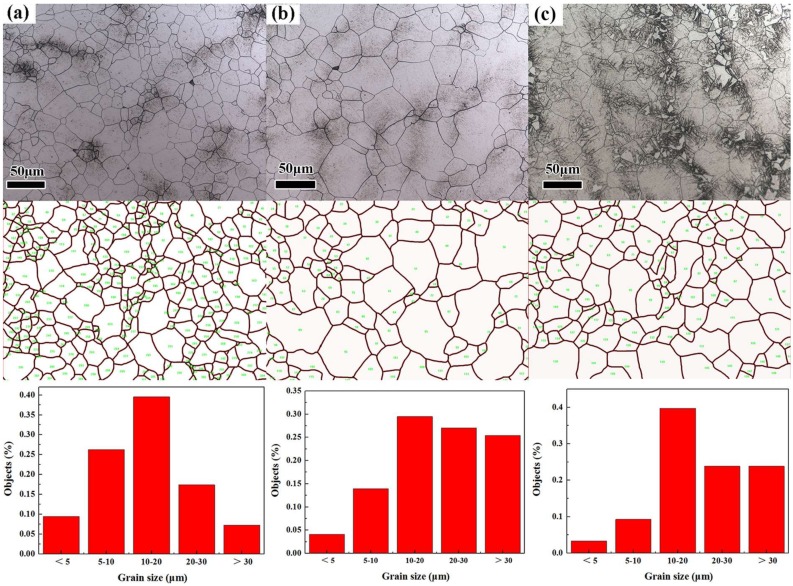
Grain micrograph of the quenched samples after different heat treatments (**a**) N-QT, (**b**) QT, and (**c**) A-T.

**Figure 8:**
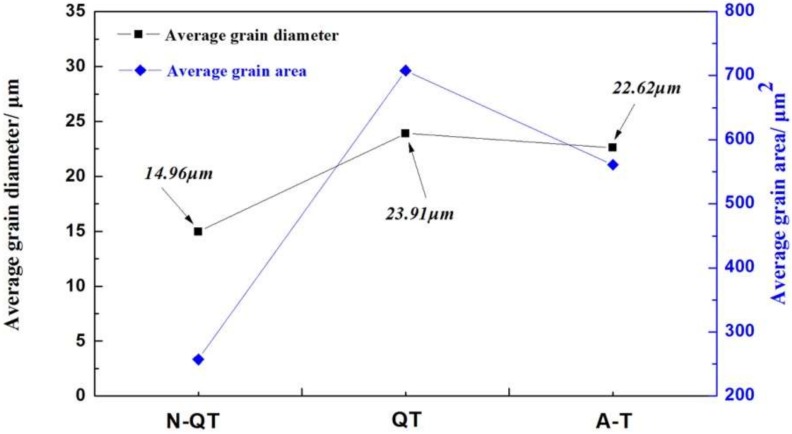
Average grain diameter and grain area of the quenched samples after different heat treatments.

**Figure 9:**
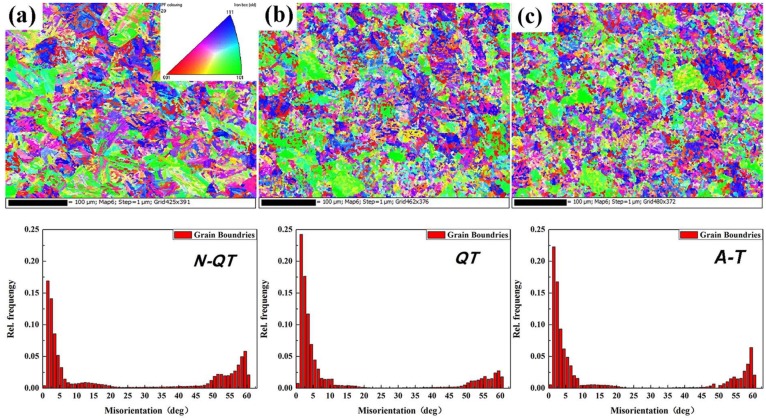
EBSD images and misorientations of the quenched samples after different heat treatments (**a**) N-QT, (**b**) QT, and (**c**) A-T.

**Figure 10:**
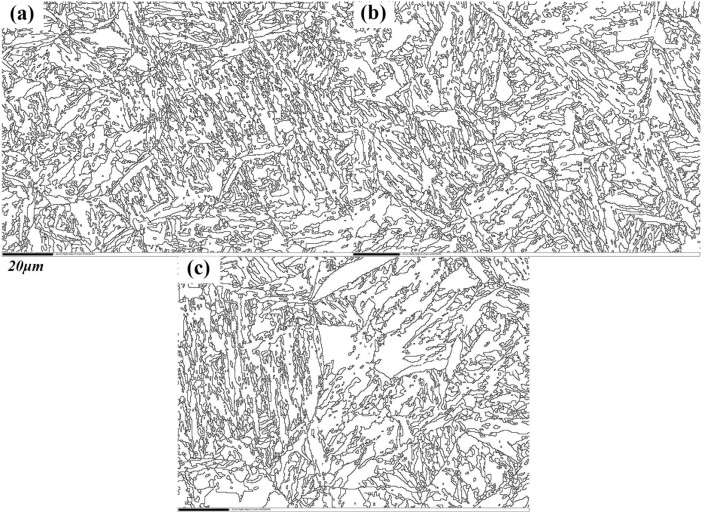
EBSD crystallographic analyses of the tempered samples after different heat treatments; analyzed area of 70 × 50 µm^2^; step size = 0.15 µm (**a**) N-QT, (**b**) QT, and (**c**) A-T. The black lines represent high-angle grain boundaries (misorientation ≥15°).

In Figure 7, the microstructures observed correspond to prior austenite grains. Quantitative relationships between the different heat treatments and the prior austenite grain size of the quenched samples is shown in Figure 8. The grain size of the quenched samples after normalizing (N-QT) significantly decreases from 23.91 to 14.96 μm compared with that of QT specimen. In comparison, the grain refinement effect of A-T specimen is relatively poor (23.91 to 22.62 μm). In addition, the typical orientation imaging maps and grain misorientation angle distributions after different heat treatments are revealed by EBSD, as shown in Figure 9. The following data analyses are average values from three different visual fields of the quenched samples. The fraction of the grain misorientation angle greater than 15° (high-angle grain boundary) is as high as 44% after N-QT treatment. Similarly, the fraction after A-T treatments is 30%. However, after QT treatment steel, it is only 23%. In addition, Figure 10 provides EBSD crystallographic analyses of the tempered samples after different heat treatments (analyzed area of 70 × 50 µm^2^; step size = 0.15 µm). We only display the high angle grain boundaries (the black lines). In our study, the effective grain boundaries were defined where the misorientation exceeds 15° by EBSD crystallographic analyses, and the effective grain size was measured using the mean linear intercept method [20]. The average grain size is determined as 1.31, 1.52, and 1.45 µm corresponding heat treatment is N-QT, QT, and A-T.

**Figure 11:**
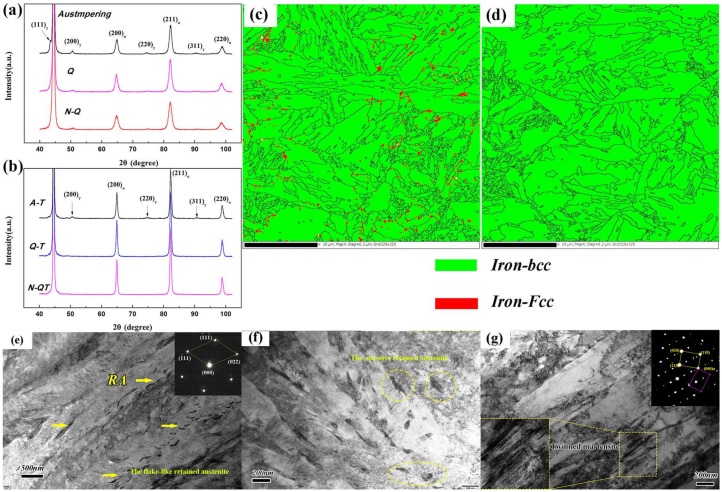
(**a**) XRD spectra of the quenched samples after different heat treatments, (**b**) XRD spectra of the tempered samples after different heat treatments, (**c**) Phase maps showing retained austenite of the tempered samples in A-T, (**d**) Phase maps showing retained austenite of the tempered samples in QT, (**e**,**f**) TEM micrographs showing the quenched microstructures of the A-T specimens, and (**g**) TEM micrographs showing the tempered microstructures of the A-T specimens. (The insert in Figure 11e is the SAED pattern of retained austenite, and the insert in Figure 11g is the martensite with a typical body-centered cubic (bcc) twin reciprocal lattice).

Figure 11a shows the XRD spectra of the quenched samples after different heat treatments tests. From these XRD spectra, the values of *V_RA_* of the quenched samples are determined as 4.58, 4.65, and 6.54 vol % when the corresponding heat treatment is N-QT, QT, and A-T, respectively. In addition, Figure 11b shows the XRD spectra of the tempered samples after different heat treatment tests. The result indicates that the value of *V_RA_* of the tempered sample is 3.93 vol % after A-T heat treatment. There is no austenite peak observed in the other specimens (N-QT and QT heat treatments). The *V_RA_* are below the detection limit which indicates a very low volume fraction of retained austenite [21]. In addition, the morphology of retained austenite in the tempered samples in A-T and QT were investigated with the phase maps by EBSD, as shown in Figure 11c (A-T) and d (QT). Austenite appears in red and the others are martensite matrix. From the comparison of the phase maps (Figure 11c,d), more film-like and massive retained austenite are clearly seen at lath boundaries after the A-T treatment compared with that of QT specimen. In addition, more details in quenching and tempering microstructures of the A-T specimens are characterized by the TEM bright-field (BF) observation and selected area electron diffraction (SAED) patterns, as shown in Figure 11e–g. The film (flake)-like (Figure 11e) and massive (Figure 11f) retained austenite are clearly found between the laths of the quenched microstructures. In addition, the tempered microstructure in Figure 11g illustrates the martensite with a typical body-centered cubic (bcc) twin reciprocal lattice.

### 3.3. Mechanical Properties

**Figure 12:**
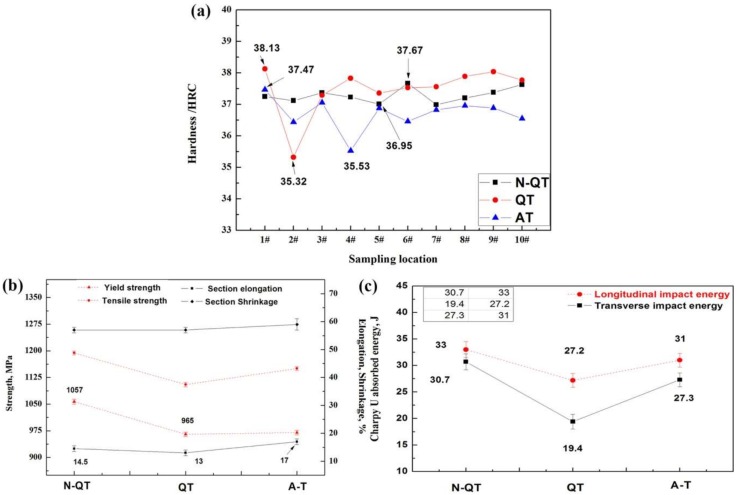
(**a**) Hardness value of samples with different heat treatments, (**b**) Effects of different heat treatments on yield, tensile strength, section shrinkage, elongation, and (**c**) Variation in impact energy of samples in response to different heat treatments.

Hardness measurements were performed on samples after different heat treatments, as shown in Figure 12a. The result indicates that the total deviation is no larger than 0.72 HRC after N-QT treatments. The small deviation of the hardness value reflects the uniformity of the tempered microstructure. However, the sample after A-T treatment exhibits poorer hardness uniformity with a total deviation greater than 1.94 HRC. Worst hardness uniformity of the sample appears after QT treatment, and the total deviation is 2.81 HRC. The tensile tests were conducted for the tempered samples after different heat treatments, and the result is shown in Figure 12b. It indicates that the tensile and yield strength of transverse samples after QT treatment are 1105 and 965 MPa, respectively. However, after N-QT treatment, they increase to 1194 and 1056 MPa, respectively. Similarly, the tensile and yield strength slightly increase after A-T treatment, and they are 1150 and 970 MPa, respectively. In addition, the sample after A-T treatment exhibits relatively higher elongation (17%) compared to the samples after N-QT (14.5%) and QT (13%) treatments. Hence, the product of strength and elongation (PSE) improved from 14.36 to 20.29 GPa% after A-T treatment. The PSE reflects the energy absorption during deformation [22].

In addition, the impact energy versus different heat treatments of the test steels is shown in Figure 12c. The result demonstrates that the impact energy of transverse samples after N-QT heat treatment significantly increases from 19.4 to 30.7 J compared with that of QT specimen. Similarly, the impact energy increases to 27.3 J after A-T treatment. The similar trend also occurs for the impact energy of longitudinal samples.

**Figure 13:**
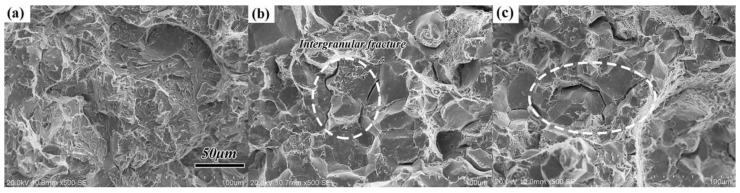
SEM micrographs of the impact fracture after different heat treatments (**a**) N-QT, (**b**) QT, and (**c**) A-T.

**Figure 14:**
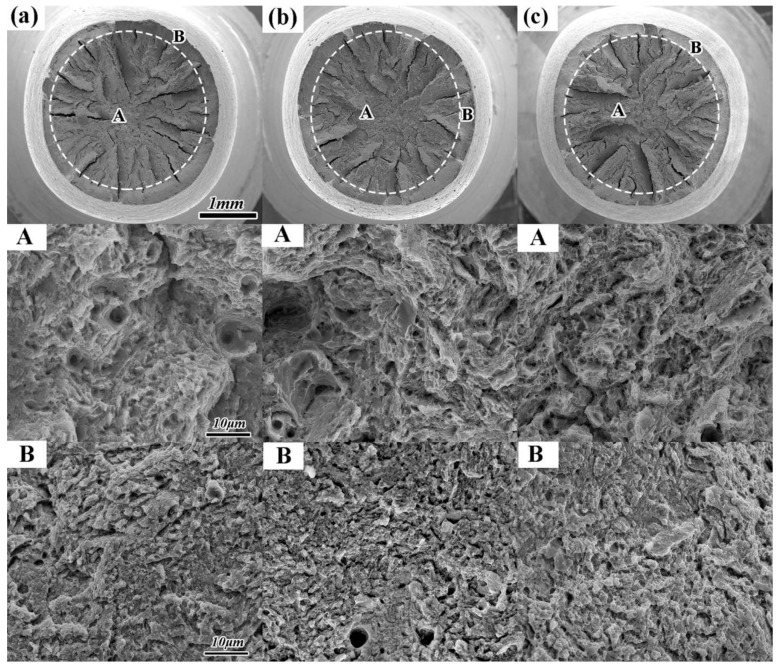
SEM micrographs of the tensile fracture after different heat treatments (**a**) N-QT, (**b**) QT, and (**c**) A-T. (A and B are the A region and B region morphology of the tensile fracture after different heat treatment, respectively).

The fracture analysis of transverse impact samples after different heat treatments are performed to reveal the morphologies of impact fracture, and only the results of a representative sample are displayed (Figure 13). The morphologies of impact fracture demonstrate brittle fracture after N-QT treatment (Figure 13a). However, the fracture morphologies of the specimens are dominated by brittle intergranular failure after QT and A-T treatments, as shown in Figure 13b,c. In addition, Figure 14 exhibits SEM micrographs of transverse tensile samples after different heat treatments. It indicates that the fracture morphology and the area fractions of A and B region for all samples after different heat treatments are similar. The fracture surface morphology consists of a wide range of different-sized dimples, which demonstrated ductile fracture. In addition, the morphology of B region in Figure 14b is presented by dimples and some voids which caused by the precipitated carbides dropping from matrix (QT). This is consistent with the description of the large size carbides easily falling off from the matrix in the literature [23].

## 4. Discussion

### 4.1. Hardness Uniformity

The hardness uniformity after N-QT treatment is better than QT treatment because the fluctuation of the cross-section hardness is 0.72 and 2.81 HRC, respectively. The worst hardness uniformity of QT specimens is mainly caused by the uneven distribution of the tempered carbides induced by the element segregation (Cr, Mo, and C) during the solidification of the test steel (Figure 2c–f). The aggregation of a higher amount of coarse carbides in the matrix is clearly seen in Figure 3e. In addition, compared with N-QT specimen, A-T specimen also exhibits poorer hardness uniformity with a total deviation greater than 1.94 HRC. This is mostly caused by the difference in microhardness between lower bainite and tempered martensite (Figure 6b). In addition, the thermal-induced martensite (Figure 11g) and retained austenite in the tempered microstructure also have a negative effect on the microhardness uniformity of A-T specimen. The fluctuations of microhardness show inhomogeneity of the cross-section hardness, which would affect the subsequent mechanical properties of the specimens [6].

### 4.2. Strengthening Mechanism

For the tempered martensite/ferrite microstructure, without taking precipitation strengthening into account, the yield strength can be expressed as Equation (2) [20,24,25,26]:

(2) $$\sigma_{YS}=\Delta \sigma_{0}+\Delta \sigma_{ss}+\Delta \sigma_{GB}+\Delta \sigma_{DIS}=88+\left(32.34\left[Mn\right]+83.16\left[Si\right]+360\left[C\right]+33\left[Ni\right]+11\left[Mo\right]+354.2\left[N\right]\right)+K_{HP}\cdot d^{-1/2}+\alpha MGb\rho^{1/2}$$

**Figure 15:**
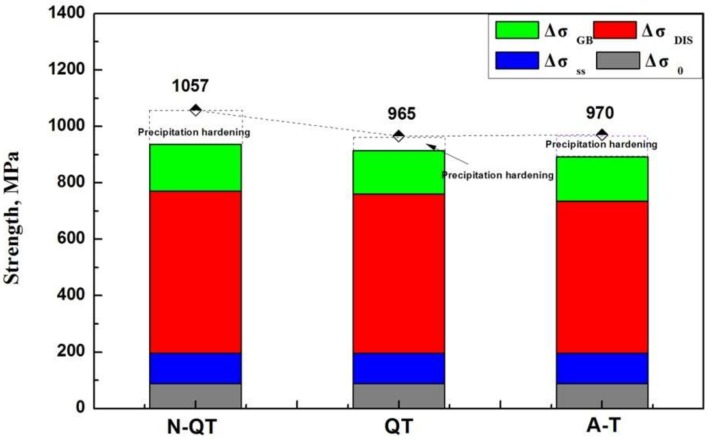
Components of the yield strength of the test steels after different heat treatments.

Here, $\sigma_{YS}$ is the yield strength of the test steel (MPa); $\Delta \sigma_{0}$ is the intrinsic strength of the matrix (MPa), which was estimated to be 85~88 MPa [27,28]; $\Delta \sigma_{ss}$ is the solid-solution strengthening (MPa), and [*Mn*], [*Si*], [*C*], [*Ni*], [*Mo*], and [*N*] is the mass fraction of elements in solution in test steel, respectively (wt %). Due to the low solubility of carbon in the high-temperature tempered martensite matrix, the contribution of carbon and carbide forming elements are considered to be negligible [28,29]; $\Delta \sigma_{GB}$ is the grain boundary strengthening (MPa), and *d* is the grain diameter (mm); for lath martensite containing 0.4 wt % C, Daigne et al. [30] showed that the grain boundary effect was determined by either the lath width or the packet size, depending on the form of carbides present. In addition, the value of $K_{HP}$ is 0.19 MPa m^−1/2^ according to the study of strengthening mechanism of the tempered martensite in medium carbon steels by Kim [29]. In addition, it is also in agreement with the values 0.21 and 0.20 MPa m^−1/2^ reported by Shibata [31] for block boundaries, and Wang [32] for martensite packets, respectively. $\Delta \sigma_{DIS}$ is the dislocation strengthening (MPa), and *M* is the Taylor factor of 3.0, *α* is a constant of 0.4, *G* is the shear modulus of 78.5 GPa, *b* is the Burgers vector of 0.248 nm, and *ρ* is the average dislocation density of the test steel, which is estimated to be 6.06 × 10^14^, 5.83 × 10^14^, and 5.34 × 10^14^ m^−2^ corresponding to N-QT, QT, and A-T heat treatments by XRD combined with MDI Jade 6.5 software. The average dislocation density in matrix for A-T specimen is less than that of N-QT and QT specimen, which is attributed to the transfer of carbon content from the matrix to the retained austenite during bainite transformation, accompanying the reduction of dislocation density [33]. Therefore, the components of the yield strength of the test steels after different heat treatments can be estimated, as shown in Figure 15.

The result shows that the main difference in the enhancement contribution are $\Delta \sigma_{GB}$ and $\Delta \sigma_{DIS}$ for different heat treatment samples. After N-QT treatment, $\Delta \sigma_{GB}$ and $\Delta \sigma_{DIS}$ are 166 and 575 MPa, respectively, however, after QT treatment, they are only 154 and 564 MPa. In addition, it indicates that there is a gap between the value of ($\Delta \sigma_{GB}$ + $\Delta \sigma_{ss}$ + $\Delta \sigma_{0}$ + $\Delta \sigma_{DIS}$) and the actual yield strength. Therefore, the precipitation strengthening is considered to influence the yield strength of the test steel.

The coarse chain carbide is a priority location for crack initiation and may lead to a dimpled fracture surface morphology (Figure 14b). The carbide precipitation is mainly distributed on the plate boundary and dislocation clusters, resulting in a strengthening effect for tempered martensite steel [11]. However, the brittle microcracks tend to grow and spread around coarse chain carbides, resulting in crack propagation [34]. The morphology and volume fraction of the tempered carbides play a critical role in improving the strength properties of test steel. Derivation of the formulas for different strength increments was based on the Ashby-Orowan model [35,36,37]. The model illustrates that the precipitation strengthening effect is proportional to *f*^1/2^ and is inversely proportional to *d*, when the slip dislocation bypasses the non-deformable particles in the Ashby-Orowan mechanism, as shown in Equation (3):

(3) $$\Delta \sigma_{p}=8.995\times 10^{3}\times \frac{f^{1/2}}{d}\times ln\left(2.417\times d\right)\;\;$$

Here, $\Delta \sigma_{p}$ is the precipitation strengthening (MPa), *f* is the volume fraction of particles, and *d* is the spatial diameter of the particles (mm). The samples subjected to the N-QT heat treatment showed much finer and spheroidized carbides (Figure 3d). However, the coarse chain carbides are unevenly distributed in the QT specimen (Figure 3e). For A-T specimen, the tensile and yield strength slightly increases compared with that of QT specimen. The strength of the tempered martensite and lower bainite is determined by the effect of various intrinsic components. According to the literature [13], the lower bainite showed a higher toughness and tensile strength than tempered martensite at equivalent hardness. In addition, the A-T specimen shows relatively a higher elongation (17%) compared with the samples after N-QT (14.5%) and QT (13%) treatments. The mechanism for enhancing the ductility after A-T treatment may be summarized as the retained austenite in matrix after tempering. Usually, the film-like and massive retained austenite is commonly observed at austenite grain boundaries [38,39,40,41]. In addition, the nucleation sites of retained austenite might tend to be changed from austenite grain boundaries to lath boundaries due to the decrease in transformation resistance [42]. The film-like retained austenite at lath boundary puts the martensite in a softening state during the deformation. The deformation ability of the martensite during uniform deformation stage is improved effectively, resulting in improvement of the ductility obviously. In addition, the film-like retained austenite between martensite is more stable than massive retained austenite as mentioned in previous literatures [43,44]. The deformation-induced martensite transformation from the unstable massive retained austenite would occur when the critical stress caused by the high density of the dislocation in some areas reach a certain critical value and lead to the Transformation Induced Plasticity (TRIP) effect. The TRIP effect would avoid the formation of cracks and delay the necking up during tensile test [45,46]. The mechanism of enhancing ductility mentioned above is suitable for the A-T specimen during tensile test because of the higher content of retained austenite of the tempered sample (3.93 vol %, Figure 11b). However, TRIP effect has not fully contributed to the ductility of A-T steel because of the formation of deformation-induced twined martensite [33]. Generally, we think that the twined martensite can prevent dislocation movement during deformation, but the dislocations accumulation will cause the stress concentration, resulting in giving priority to crack formation [47]. Simultaneously, the crack can easily propagate through deformation-induced martensite with a straight propagation path [48], which led to a significant decrease in strength and ductility. Although the martensite transformation caused by deformation-induced can absorb energy, it may occur before the crack tips of high stress concentration. However, on the contrary, some novel studies showed that the twined martensite enhanced the strength without reducing plasticity if it had nano size and was in very small amounts [49,50].

Simultaneously, the slightly thermal-induced twined martensite is found to be embedded in the matrix in the A-T tempered specimen (Figure 11g). Qin [33] and Podder [51] as well as Wang [52] also confirmed the twinned martensite transformation from high-carbon austenite during tempering. The high-carbon massive retained austenite becomes unstable due to the partial decrease of carbon concentration after the carbides precipitation during tempering, resulting in transformation of retained austenite into twinned martensite. Therefore, it can be summarized as that the stable film-like retained austenite can effectively prevent crack propagation, which leads to the increase of crack propagation energy. However, the unstable massive retained austenite can easily be converted into twined martensite during tempering and subsequent tensile process. Therefore, the ductility of the A-T specimen mainly depends on the amount and morphology of retained austenite and thermal/deformation-induced twined martensite.

### 4.3. Impact Toughness

A major factor for the increase in the impact energy after N-QT heat treatment is the more high-angle grain boundaries (the grain misorientation angle greater than 15°) compared with that of QT specimen (Figure 9). The reference also indicated that the crystal orientation and crack direction could change when a crack attempted to propagate across a high-angle boundary, resulting in delaying of the final crack propagation [53]. In addition, another investigation showed that the original austenite grain boundary is the high-angle grain boundary, while the lath martensite boundary is the low-angle grain boundary [54]. The greater the number of high-angle grain boundaries, the smaller the effective grain size of the microstructure, resulting in absorbing more energy during crack propagation [55]. Therefore, the increase in the number of high-angle boundaries would lead to more deflection of the crack and more favorable toughness. For the A-T specimen, the transverse impact energy increases to 27.3 J compared with that of QT specimen (19.4 J). On the one hand, that can be explained by a small amount of increase of the high-angle grain boundary (Figure 9). On the other hand, it can be explained by the volume fraction of retained austenite of the tempered sample at lath boundaries (Figure 11d). According to research by Zhang [21] and Wang [56], stable retained austenite can absorb dislocations from the adjacent lath martensite continuously and can effectively blunt crack propagation.

In addition, the fracture surface of longitudinal impact samples after QT and A-T treatments are dominated by brittle intergranular failure, as shown in Figure 13b,c. The reason can be attributed to grain boundary weakening. The smaller the grain size, the larger the total grain boundary area per unit volume. Grain boundary area per unit volume, $S_{v}$, can be expressed as a function of mean interception length of grains (mean grain size), *d*, as Equation (4) [57]

(4) $$S_{v}=\frac{2}{d}$$

Therefore, the grain boundary area per unit volume in the specimens after N-QT, QT, and A-T treatments are 1.33 × 10^5^ m^2^/m^3^ (N-QT), 8.3 × 10^4^ m^2^/m^3^ (QT), and 8.8 × 10^4^ m^2^/m^3^ (A-T), respectively. Consequently, in the grain boundary area per unit volume after N-QT treatment, the concentration of the hydrogen or other harmful elements (phosphorus, sulfur and so on) that weaken the resistance of grain boundaries decreases with the decrease of the grain size. As a result, the resistance of grain boundaries increases, and the occurrence of intergranular fracture reduces.

### 4.4. Selection of Optimal Heat Treatment Process

Based on the actual industrial application requirements, the room temperature Charpy U-notch impact energy, tensile strength, yield strength, and fluctuations of hardness of large cross-section 718H pre-hardened mold steel are minimally 20 J/cm^2^, 1100 MPa, 980 MPa, and 2 HRC, respectively [11]. In addition, no retained austenite is expected to be present in the product of 718H pre-hardened mold steel, as martensite transformation caused by stress-strain would deteriorate the machining properties as well as the dimensional accuracy of the mold [58]. Combined with the actual industrial application requirements and the amount of retained austenite of the test steel, the optimal heat treatment process for industrial applications could be determined as normalizing-quenching-tempering (N-QT) treatment.

## 5. Conclusions

The strength-toughness combination and hardness uniformity in large cross-section 718H pre-hardened mold steel were investigated with different heat treatments for industrial applications. The following conclusions could be drawn:

(1) The specimens after N-QT treatment possessed the best strength-toughness combination and hardness uniformity compared with that of A-T and QT treatment. The microscopic element segregation, grain structure, the dislocation density, the precipitation carbides, and retained austenite were all responsible for the change of mechanical properties.
(2) The A-T treatment specimen possessed the worse hardness uniformity with a total deviation greater than 1.94 HRC compared with N-QT treatment. It was mostly attributed to the gap of microhardness between lower bainite and tempered martensite. In addition, the twinned martensite and retained austenite in the tempered microstructure also had a negative effect on the microhardness uniformity.
(3) The A-T treatment specimen showed relatively higher elongation (17%) compared with the specimens after N-QT (14.5%) and QT (13%) treatments. The ductility of A-T specimen mainly depended on the amount and morphology of retained austenite and thermal/deformation-induced twined martensite.
(4) The reason for brittle intergranular failure of the impact specimens after QT and A-T treatments could be attributed to grain boundary weakening.

Combined with the actual industrial application requirements and the amount of retained austenite of the test steel, the optimal heat treatment process could be determined as N-QT treatment. This work presents an effective heat treatment route to improve strength-toughness-hardness trade-off in large cross section steels.

## Figures and Tables

**Table 1:** Chemical compositions at different positions of test 718H steel (wt %).

C	Si	Mn	Cr	Ni	Mo	P	S	Al
0.34	0.33	1.56	2.06	1.04	0.20	0.016	0.005	0.017

## References

[1] Wu X., Xu L. (2011). New Products and Techniques of Mould Steels.

[2] Wu R., Li J., Su Y., Liu S., Yu Z. (2017). Improved uniformity of hardness by continuous low temperature bainitic transformation in prehardened mold steel with large section. Mater. Sci. Eng. A.

[3] Wu R.M., Zheng Y.F., Wu X.C., Li X.C. (2016). Effect of titanium on the microstructure and hardness uniformity of non-quenched and tempered prehardened steel for large-section plastic mould. Ironmak. Steelmak..

[4] González H., Calleja A., Pereira O., Ortega N., Norberto López de Lacalle L., Barton M. (2018). Super Abrasive Machining of Integral Rotary Components Using Grinding Flank Tools. Metals.

[5] Hoseiny H., Högman B., Klement U., Kinnander A. (2012). Machinability evaluation of prehardened plastic mould steels. Int. J. Mach. Mach. Mater..

[6] Hoseiny H., Caballero F.G., Saoubi R.M., Högman B., Weidow J., Andrén H.O. (2015). The Influence of Heat Treatment on the Microstructure and Machinability of a Prehardened Mold Steel. Metall. Mater. Trans. A.

[7] Luo Y., Wu X.C., Wang H.B., Min Y.A. (2009). A comparative study on non-quenched and quenched prehardened steel for large section plastic mould. J. Mater. Process. Technol..

[8] Firrao D., Matteis P., Spena P.R., Gerosa R. (2013). Influence of the microstructure on fatigue and fracture toughness properties of large heat-treated mold steels. Mater. Sci. Eng. A.

[9] Firrao D., Matteis P., Scavino G., Ubertalli G., Ienco M.G., Pinasco M.R., Stagno E., Gerosa R., Rivolta B., Silvestri A. (2007). Relationships between tensile and fracture mechanics properties and fatigue properties of large plastic mould steel blocks. Mater. Sci. Eng. A.

[10] Hoseiny H., Klement U., Sotskovszki P., Andersson J. (2011). Comparison of the microstructures in continuous-cooled and quench-tempered pre-hardened mould steels. Mater. Des..

[11] Liu H.H., Fu P.X., Liu H.W., Sun C., Ma X.P., Li D.Z. (2018). Microstructure evolution and mechanical properties in 718H pre-hardened mold steel during tempering. Mater. Sci. Eng. A.

[12] Wen X.L., Mei Z., Jiang B., Zhang L.C., Liu Y.Z. (2016). Effect of normalizing temperature on microstructure and mechanical properties of a Nb-V microalloyed large forging steel. Mater. Sci. Eng. A.

[13] Tu M.Y., Hsu C.A., Wang W.H., Hsu Y.F. (2008). Comparison of microstructure and mechanical behavior of lower bainite and tempered martensite in JIS SK5 steel. Mater. Chem. Phys..

[14] Kim S., Lee Y. (2011). Effect of retained austenite phase on springback of cold-rolled TRIP steel sheets. Mater. Sci. Eng. A.

[15] Couturier L., Geuser F.D., Descoins M., Deschamps A. (2016). Evolution of the microstructure of a 15-5PH martensitic stainless steel during precipitation hardening heat treatment. Mater. Des..

[16] Dijk N.H.V., Butt A.M., Zhao L., Sietsma J., Offerman S.E., Wright J.P., Zwaag V.D. (2005). Thermal stability of retained austenite in TRIP steels studied by synchrotron X-ray diffraction during cooling. Acta Mater..

[17] Li X.L., Wang Z.D. (2015). Effect of one step Q&P process on microsturcture and mechanical properties of a dual martensite steel. Acta Metall. Sin..

[18] Bhadeshia H.K.D.H. (2001). Bainite in Steels: Transformation, Microstructure and Properties.

[19] Dong J., Zhou X., Liu Y., Li C., Liu C., Guo Q. (2017). Carbide precipitation in Nb-V-Ti microalloyed ultra-high strength steel during tempering. Mater. Sci. Eng. A.

[20] Chen J., Zhang W., Liu Z., Wang G. (2017). The Role of Retained Austenite on the Mechanical Properties of a Low Carbon 3Mn-1.5Ni Steel. Metall. Mater. Trans. A.

[21] Zhang K., Zhang M., Guo Z., Chen N., Rong Y. (2011). A new effect of retained austenite on ductility enhancement in high-strength quenching–partitioning–tempering martensitic steel. Mater. Sci. Eng. A.

[22] Gao G., An B., Zhang H., Guo H., Gui X., Bai B. (2017). Concurrent enhancement of ductility and toughness in an ultrahigh strength lean alloy steel treated by bainite-based quenching-partitioning-tempering process. Mater. Sci. Eng. A.

[23] Zhang J., Wang F.M., Yang Z.B., Li C.R. (2016). Microstructure, Precipitation, and Mechanical Properties of V-N-Alloyed Steel After Different Cooling Processes. Metall. Mater. Trans. A.

[24] Chen J., Lv M.Y., Tang S., Liu Z.Y., Wang G.D. (2014). Influence of cooling paths on microstructural characteristics and precipitation behaviors in a low carbon V–Ti microalloyed steel. Mater. Sci. Eng. A.

[25] Bouquerel J., Verbeken K., Cooman B.D. (2006). Microstructure-based model for the static mechanical behaviour of multiphase steels. Acta Mater..

[26] Yen H.W., Chen P.Y., Huang C.Y., Yang J.R. (2011). Interphase precipitation of nanometer-sized carbides in a titanium–molybdenum-bearing low-carbon steel. Acta Mater..

[27] Halfa H. (2014). Recent Trends in Producing Ultrafine Grained Steels. J. Miner. Mater. Charact. Eng..

[28] Cheng X.Y., Zhang H.X., Li H., Shen H.P. (2015). Effect of tempering temperature on the microstructure and mechanical properties in mooring chain steel. Mater. Sci. Eng. A.

[29] Kim B., Boucard E., Sourmail T., Martín D.S., Gey N., Rivera-Díaz-del-Castillo P.E.J. (2014). Understanding the microstructure–properties relationship in 0.5–0.6 wt % C steels. Acta Mater..

[30] Daigne J., Guttmann M., Naylor J.P. (1982). The influence of lath boundaries and carbide distribution on the yield strength of 0.4% C tempered martensitic steels. Mater. Sci. Eng..

[31] Shibata A., Nagoshi T., Sone M., Morito S., Higo Y. (2010). Evaluation of the block boundary and sub-block boundary strengths of ferrous lath martensite using a micro-bending test. Mater. Sci. Eng. A.

[32] Wang J.S., Mulholland M.D., Olson G.B., Seidman D.N. (2013). Prediction of the yield strength of a secondary-hardening steel. Acta Mater..

[33] Qin S., Liu Y., Hao Q., Wang Y., Chen N.L., Zuo X.W., Rong Y.H. (2015). The Mechanism of High Ductility for Novel High-Carbon Quenching–Partitioning–Tempering Martensitic Steel. Metall. Mater. Trans. A.

[34] Chen J.D., Mo W.L., Wang P., Lu S.P. (2012). Effects of tempering temperature on the impact toughness of steel 42CrMo. Acta Metall. Sin..

[35] Soto R., Saikaly W., Bano X., Issartel C., Rigaut G., Charai A. (1999). Statistical and theoretical analysis of precipitates in dual-phase steels microalloyed with titanium and their effect on mechanical properties. Acta Mater..

[36] Gladman T., Dulieu D., Mivor I.D. (1977). Proc. Int. Conf. on High Strength Low Alloy Steels—Microalloying 75.

[37] Cao J.C., Yong Q.L., Liu Q.Y., Sun X. (2007). Precipitation of MC phase and precipitation strengthening in hot rolled Nb–Mo and Nb–Ti steels. J. Mater. Sci..

[38] Gao G.H., Zhang H., Gui X.L., Luo P., Tan Z.L., Bai B.Z. (2014). Enhanced ductility and toughness in an ultrahigh-strength Mn–Si–Cr–C steel: The great potential of ultrafine filmy retained austenite. Acta Mater..

[39] Nakada N., Mizutani K., Tsuchiyama T., Takaki S. (2014). Difference in transformation behavior between ferrite and austenite formations in medium manganese steel. Acta Mater..

[40] Chen J., Lv M.Y., Tang S., Liu Z., Wang G. (2015). Correlation between mechanical properties and retained austenite characteristics in a low-carbon medium manganese alloyed steel plate. Mater. Charact..

[41] Chen J., Lv M.Y., Liu Z.Y., Wang G.D. (2016). Influence of Heat Treatments on the Microstructural Evolution and Resultant Mechanical Properties in a Low Carbon Medium Mn Heavy Steel Plate. Metall. Mater. Trans. A.

[42] Nakada N., Tsuchiyama T., Takaki S., Miyano N. (2011). Temperature Dependence of Austenite Nucleation Behavior from Lath Martensite. ISIJ Int..

[43] Xiong X.C., Chen B., Huang M.X., Wang J.F., Wang L. (2013). The effect of morphology on the stability of retained austenite in a quenched and partitioned steel. Scr. Mater..

[44] Li Y.J., Li X.L., Yuan G., Kang J., Chen D., Wang G.D. (2016). Microstructure and partitioning behavior characteristics in low carbon steels treated by hot-rolling direct quenching and dynamical partitioning processes. Mater. Charact..

[45] Slycken J.V., Verleysen P., Degrieck J., Samek L., Cooman B.C.D. (2006). High-strain-rate behavior of low-alloy multiphase aluminum- and silicon-based transformation-induced plasticity steels. Metall. Mater. Trans. A.

[46] Curtze S., Kuokkala V.T., Hokka M., Peura P. (2009). Deformation behavior of TRIP and DP steels in tension at different temperatures over a wide range of strain rates. Mater. Sci. Eng. A.

[47] Hao Q., Qin S., Liu Y., Zuo X.W., Chen N.L., Huang W., Rong Y.H. (2016). Effect of retained austenite on the dynamic tensile behavior of a novel quenching-partitioning-tempering martensitic steel. Mater. Sci. Eng. A.

[48] Sohn S.S., Hong S., Lee J., Sun B.C., Kim S.K., Lee B.J., Kim N.J., Lee S. (2015). Effects of Mn and Al contents on cryogenic-temperature tensile and Charpy impact properties in four austenitic high-Mn steels. Acta Mater..

[49] Seo E.J., Cho L., Estrin Y., Cooman B.C.D. (2016). Microstructure-mechanical properties relationships for quenching and partitioning (Q&P) processed steel. Acta Mater..

[50] Li Y.J., Kang J., Zhang W.N., Liu D., Wang X.H., Yuan G., Misra R.D.K., Wang G.D. (2018). A novel phase transition behavior during dynamic partitioning and analysis of retained austenite in quenched and partitioned steels. Mater. Sci. Eng. A.

[51] Podder A.S., Bhadeshia H.K.D.H. (2010). Thermal stability of austenite retained in bainitic steels. Mater. Sci. Eng. A.

[52] Wang K., Tan Z., Gu K., Gao B., Gao G.H., Misra R.D.K., Bai B.Z. (2017). Effect of deep cryogenic treatment on structure-property relationship in an ultrahigh strength Mn-Si-Cr bainite/martensite multiphase rail steel. Mater. Sci. Eng. A.

[53] Liu J., Yu H., Zhou T., Song C., Zhang K. (2014). Effect of double quenching and tempering heat treatment on the microstructure and mechanical properties of a novel 5Cr steel processed by electro-slag casting. Mater. Sci. Eng. A.

[54] Morito S., Tanaka H., Konishi R., Furuhara T., Maki T. (2003). The morphology and crystallography of lath martensite in Fe-C alloys. Acta Mater..

[55] Guan Q., Jiang Q., Fang J., Jiang H. (2003). Microstructures and Thermal Fatigue Behavior of Cr-Ni-Mo Hot Work Die Steel Modified by Rare Earth. ISIJ Int..

[56] Wang X.D., Guo Z.H., Rong Y.H. (2011). Mechanism exploration of an ultrahigh strength steel by quenching–partitioning–tempering process. Mater. Sci. Eng. A.

[57] Bai Y., Momotani Y., Chen M.C., Shibata A., Tsuji N. (2016). Effect of grain refinement on hydrogen embrittlement behaviors of high-Mn TWIP steel. Mater. Sci. Eng. A.

[58] Krajnik P., Kopač J. (2004). Modern machining of die and mold tools. J. Mater. Process. Technol..

